# The glycocalyx as a permeability barrier: basic science and clinical evidence

**DOI:** 10.1186/s13054-022-04154-2

**Published:** 2022-09-12

**Authors:** Randal O. Dull, Robert G. Hahn

**Affiliations:** 1 Department of Anesthesiology, University of Arizona College of Medicine, Tucson, AZ USA; 2 Department of Pathology, University of Arizona College of Medicine, Tucson, AZ USA; 3 Department of Surgery, University of Arizona College of Medicine, Tucson, AZ USA; 4 Karolinska Institute at Danderyds Hospital (KIDS), Danderyd, Stockholm, Sweden

**Keywords:** Anesthesia, Capillaries, Glycocalyx, Human studies, Permeability, Fluid kinetics, Translational research

## Abstract

Preclinical studies in animals and human clinical trials question whether the endothelial glycocalyx layer is a clinically important permeability barrier. Glycocalyx breakdown products in plasma mostly originate from 99.6–99.8% of the endothelial surface not involved in transendothelial passage of water and proteins. Fragment concentrations correlate poorly with in vivo imaging of glycocalyx thickness, and calculations of expected glycocalyx resistance are incompatible with measured hydraulic conductivity values. Increases in plasma breakdown products in rats did not correlate with vascular permeability. Clinically, three studies in humans show inverse correlations between glycocalyx degradation products and the capillary leakage of albumin and fluid.

## Introduction

The endothelial glycocalyx has long been hypothesized to be a major component of the microvascular permeability barrier in humans. In fact, almost every published paper on the glycocalyx includes “permeability barrier” as one of its major functions. The data, however, for such a conclusion are scarce. The basic science and preclinical evidence for the glycocalyx as a primary permeability barrier will be reviewed.

**Fig. 1:**
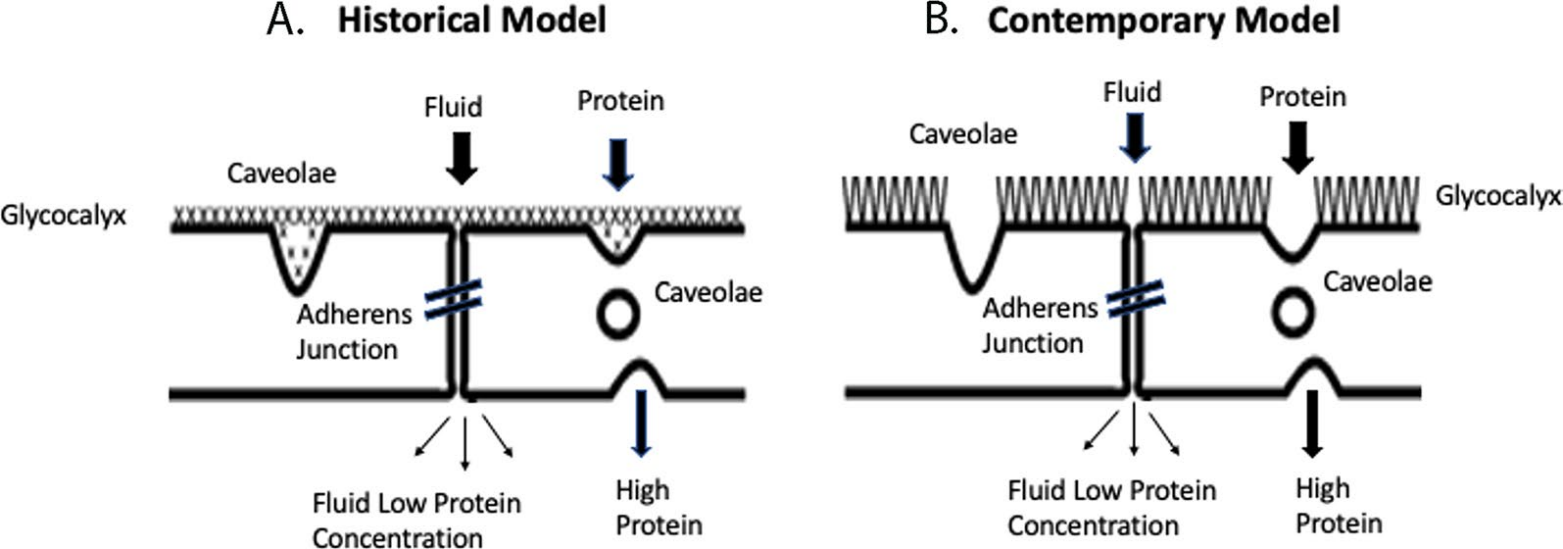
**A** Historical model of the glycocalyx (left), based on electron micrographs, proposed that the endothelial glycocalyx covered the cell surface including the luminal opening of the intercellular junction and the neck of caveolae. **B** The contemporary model (right) suggests that the glycocalyx residing over the cell body is much thicker than originally proposed but does not appreciably cover the junctional opening or caveolae neck. In the contemporary model, junctional complexes like the adherens junction limits protein permeability rather than the glycocalyx. Modified from Reference [3]

The primary pathway for fluid and solute movement across the endothelium is through the intercellular junction (Fig. 1). In mammals, the intercellular junction is a long and tortuous cleft held together by the adherens junction (AJ), a complex of proteins that create focal areas of tight membrane apposition. The AJ complex is, in turn, connected to the endothelial cytoskeleton via an array of signaling molecules and adhesion proteins that, during inflammation, can disassemble leading to an increase in junctional permeability [1]. Vesicular transport, via caveolae, participates in albumin transport in the continuous-type endothelium [2] but remains an unsettled issue in fenestrated endothelium.

Early electron micrographs yielded images of a compact endothelial surface layer, while more recent images presented a thick bush-like layer covering the cell surface. Based on these images, the glycocalyx has been widely depicted a formidable layer covering the cell surface including overlying the luminal opening of the intercellular junction and the caveolae neck. In both instances, the glycocalyx has been presumed to function as a molecular filter for junctional and vesicular transport [3–6] (Fig. 1). Regarding caveolae, recent evidence suggests that PV1, a caveolae diaphragm protein, is the principal determinant of caveolae albumin loading. Conditional knockdown of PV1 dramatically increases caveolae albumin loading and transcytosis, leading to a significant loss of plasma albumin, increased tissue albumin concentrations and extensive interstitial edema [7].

## Structural perspective

Images of the glycocalyx show a dense fuzzy layer on the endothelial surface and create the sense of a physical barrier [8–10]*.* The images presented in these references show plug-like structures of fenestrated endothelium, a type of endothelium, that have different permeability characteristic relative to continuous-type endothelium (Table 1). The photomicrographs of continuous-type endothelial glycocalyx by Okada et al. [10] show a bulbous-like structure that the authors call “broccoli-like,” but the images do not help us understand their location relative the endothelial junction. It is possible that fenestrated plugs are elements of the permeability barrier in this special endothelial type, but fenestrated endothelium is not typically representative of sites where permeability is clinically relevant.

**Table 1:** Permeability characteristics of common types of endothelium

Tissue	Endothelial type	*K*_*f*_ ml × min^−1^ mm Hg^−1^ 100 g^−1^	*L*_*p*_ cm s^−1^ dyne^−1^ × 10^10^	*σ*
Skeletal muscle	Continuous	0.04	0.55	0.91
Cardiac	Continuous	0.34	0.86	0.80
Intestine	Fenestrated	0.70	3.2	0.9
Salivary gland	Fenestrated	1.3	3.1	0.8

Based on data retrieved from references [11, 12]

Non-dehydrating techniques and use of cationic dyes that claimed to preserve structure have shown a fuzzy and filamentous layer, much less dense than early micrographs, but many more microns in height [13]. In this study, Van den Berg and colleagues reported that hyaluronidase treatment resulted in almost complete loss of the glycocalyx and was associated with interstitial edema. Their conclusion was that the glycocalyx was the main permeability barrier. An alternative explanation is that enzymatic digestion of glycocalyx components, known to be connected to receptors and cytoskeletal elements, may, via signaling mechanisms, open the cell junction.

Schmidt et al*.* [14] demonstrated that endotoxemia, in a murine model, induced glycocalyx disruption and increased lung microvascular permeability. Inhibition of heparanase attenuated the endotoxemia-induced hyperpermeability. A simple explanation could be that endogenous heparanase resulted in glycocalyx degradation that was the direct cause of hyperpermeability and heparanase inhibition protected the glycocalyx to maintain the permeability barrier. However, the authors concluded that heparanase inhibition prevented glycocalyx breakdown and reduced neutrophil adhesion to the endothelium, thus attenuating acute lung injury and the associated microvascular permeability.

The process demonstrated by Schmidt et al. was first described by Lipowsky and colleagues in a series of elegant papers (for review, see [15]). They demonstrated that the glycocalyx shields endothelial surface adhesion proteins and prevents neutrophil access to these adhesion sites. Glycocalyx shedding exposes these adhesion proteins to neutrophils rolling on the endothelial surface and ligation of neutrophil proteins with endothelial adhesion molecules stimulates the release of permeability-enhancing substances. Among these substances is CAP37/azurocidin, a potent permeability-enhancing mediator that opens endothelial junctions [16].

The thickness of the glycocalyx in electron micrographs of Van Der Berg [13] and by fluorescent imaging [20] is difficult to reconcile with the dimensions of individual proteoglycan components. Syndecan-1, a major constituent of the glycocalyx, possesses a core protein that contains approximately 252 amino acids in the extracellular domain and has glycosaminoglycan chains typically composed of 100 disaccharides. The height of the syndecan-1 core protein is less than 100 nm and, even if the glycosaminoglycan chains were oriented vertically, they would have to yield another 50 nm to reach a total height of 100–150 nm. The reported in vivo dimensions for the glycocalyx as approximately 500 nm–2 µm in thick is challenging to explain; it would require an absorbed protein layer on top of the syndecan-1 layer that is at least 4–8 times thicker than the underlying scaffolding. The glycocalyx, per se*,* at 100 nm height plus a much thicker extended surface layer of absorbed serum proteins is often called the *endothelial surface layer* [17]. These differing functional layers and nomenclatures blur the distinction between the glycocalyx and an absorbed protein layer and create challenges regarding which is the true permeability barrier.

## Physiological perspective

Ex vivo physiological studies using mesenteric microvessels of frogs suggested that the glycocalyx (referred to at the time of these studies as the fiber matrix) participated in barrier regulation based on the presumed effects of serum albumin [18]. For example, washout of proteins from the glycocalyx using protein-free Ringer’s solution resulted in a several fold increase in hydraulic conductivity (*L*_*p*_), while reperfusion with albumin-containing Ringer’s restored permeability values toward normal [19, 20]. Similar studies using dextran polymers and other serum proteins failed to show a permeability-lowering effect, suggesting that there was a specificity of albumin for the fiber matrix and the associated effects on permeability [21, 22]. Electron micrographs, in combination with microvessel perfusion studies, demonstrated that labeled cationized ferritin failed to penetrate the native glycocalyx, but when albumin was washed out of the glycocalyx by protein-free perfusate, the cationized ferritin showed deep penetration including vesicular uptake [6, 23]. Collectively, these results suggested that serum proteins and specifically, albumin, bind to the glycocalyx and enhanced barrier function by impeding penetration of other serum proteins. We now recognize that albumin can shuttle sphingosine-1-phosphate (S1P) between red blood cells and the endothelium resulting in improved barrier function. When S1P binds to the S1P-receptor on endothelial cells, it sets in motion a cascade of signaling events that enhances cortical actin and bolsters cell–cell contacts resulting in a tighter junction and reduced junctional permeability [24]. S1P has also been reported to protect the glycocalyx through inhibition of metalloproteinases that enzymatically cleave protein components of the glycocalyx [25].

Vink and Duling [26, 27] provided the first functional characterization of alleged glycocalyx barrier properties in vivo by demonstrating a red blood cell exclusion zone located between the endothelial surface and the flowing plasma layer. This exclusion zone was inaccessible to dextrans ≥ 70 kDa but accessible to fluorescently labeled proteins and dextrans < 70 kDa. Estimates of the glycocalyx thickness were approximately 0.5 µm. Subsequent studies using time-based fluorescent video microscopy measured the penetration times for a variety of dextrans and serum proteins. Neutral and anionic dextrans ≥ 70 kDa failed to penetrate the glycocalyx, while anionic dextrans smaller than 40 kDa entered the glycocalyx slowly. Somewhat puzzling was the observation that albumin (67 kDa) and fibrinogen (340 kDa) both penetrated the glycocalyx with similar rates despite widely different molecular weights. Such an observation casts doubts on the resolution and sensitivity of the imaging system.

Other challenges that arise from their data are the transendothelial permeability coefficient (*P*) for albumin, which has elsewhere been reported to be *P* = 10^–7^–10^–8^ cm/s [28]. *P* can be derived from *P* = *D*/*d*, where *D* is the diffusion coefficient in an aqueous substrate and *d* is the thickness of the layer. In Vink and Duling’s experiments, *d* = 0.4 µm and *D* = 8.5 × 10^–7^ cm^2^/s [26], which yields *P* = 2.1 × 10^–2^ cm/s. This value is clearly much higher than the measured *P* for albumin. Similarly, Gao and Lipowsky determined *D* to be 10^–9^ cm^2^/s for 70 kD dextran inside the rat capillary glycocalyx [29]. Using this value to calculate permeability (*P* = *D*/*d*) yields *P* = 10^–5^ cm/s which is 3 orders of magnitude greater than measured in vivo permeability coefficient. The simple interpretation of these data is that transendothelial permeability (*P*) is not determined by the glycocalyx, but more likely by the molecular complexes that create the endothelial junctions.

## Albumin, cell calcium and permeability

As second messengers and signaling pathways became an important focus of endothelial biology, it was natural to assess the effects of albumin on intracellular Ca^++^ and other permeability-enhancing second messengers. Surprisingly, removal of albumin from vessel perfusate leads to an increase in intra-endothelial Ca^++^, while reperfusion with albumin-containing perfusate resulted in an immediate decrease in intercellular Ca^++^. This increase in calcium was associated with an increase in endothelial permeability as assessed by measuring hydraulic conductivity [30]. Thus, it could now be argued that albumin *directly* modulates Ca^++^ homeostasis and *indirectly* affects endothelial permeability. The authors concluded that these results did not conform to the absorption model of albumin onto the glycocalyx as previously proposed in their earlier publications [20]. Almost universally, increases in endothelial cytoplasmic calcium increase permeability via contractile processes that open intercellular junctions. Thus, it is impossible to distinguish a direct effect of albumin on the glycocalyx versus its effect on modulating calcium-induced changes on junctional permeability [31, 32].

Albumin per se has a negative charge that makes passage to the interstitium more difficult. Albumin also has many non-oncotic properties that may be beneficial and even therapeutic in situations that cause endothelial dysfunction and increase endothelial permeability [33]. For example, albumin can reduce nitric oxide synthase activity [34] which is a major cause of oxidative stress during inflammation that results in increased junctional permeability. It also has antioxidant properties via its cys34 residue, which represents the largest pool of thiols in the vascular system [35]. Albumin exerts immunomodulatory effects including binding of lipopolysaccharides [36] and prevention of the adhesion of activated polymorphonuclear leukocytes on the endothelial surface [37], which are both factors known open endothelial cell junctions.

## In vivo glycocalyx and permeability in animals

The first direct evidence for the resistance to transendothelial water flux offered by the in vivo glycocalyx came from Adamson et al*.* [38] who used pronase, a broad-spectrum protease, to digest the endothelial surface of perfused frog microvessels while measuring hydraulic conductivity (*L*_*p*_). Degradation of the glycocalyx was assessed by quantifying cationized ferritin binding to the endothelial surface. Pronase increased *L*_*p*_ by approximately 2.5 × and reduced cationized ferritin binding by 50%. Pronase, however, is a mixture of broad-spectrum proteases and is expected to cleave junctional proteins. The authors performed electron microscopic evaluation of junctional architecture and failed to demonstrate junctional changes consistent with the increase in *L*_*p*_. Significant degradation of the glycocalyx resulted in only a modest increase in measured permeability, but the effects of pronase on junctional patency could not be excluded.

Huxley and Williams [39] reported that degradation of the glycocalyx of porcine coronary arterioles with pronase or heparinase doubled the permeability-surface area coefficient (PS) for albumin and lactalbumin. However, their subsequent analysis failed to demonstrate the expected effects of enzyme-induced permeability changes when fit to a model of resistors in series (glycocalyx, endothelial cell, arteriolar wall). Briefly, the series resistor model proposed by Huxley et al. was: *R*_total_ = *R*_glycocalyx_ + *R*_arterial wall_. Per the methods of the Huxley paper, permeability (*P*) in untreated vessels would be given by (*P*_total_)^−1^ = (*P*_glycocalyx_)^−1^ + (*P*_wall_)^−1^. By measuring permeability in untreated vessels and enzyme-treated vessels, the permeability of the glycocalyx could be derived. Using their data, the authors conclude “the logical predictions of a series resistor model failed to account for the data of the present study.” (Please see reference 38 for greater detail.) The authors provided alternative explanations including: (1) Pronase affected both junctional permeability, and the glycocalyx and (2) the model using series resistors was wrong. To these alternatives explanations, we can add that the glycocalyx might not be a major component of the permeability barrier.

This brings us to the crux of the argument against the glycocalyx being a major component of the permeability barrier. The only portion of the glycocalyx capable of influencing permeability is the fraction overlying the opening of cell–cell junctions. This is based on the following thought process: The primary pathway across the endothelium for the transport of water and solutes is through the endothelial junction. The resistance to fluid and solute movement through the junctional cleft is the overlying glycocalyx and the junctional proteins inside the cleft that hold the two adjacent endothelial cells in close apposition to each other*.* Based on detailed measurements of junctional dimensions and measured permeability coefficients, Weinbaum et al*.* [40] concluded that the glycocalyx over the junctional opening must be absent or structurally different than the thick layer found over the cell body to account for measured transport parameters.

Michel and Curry [3] provided a quantitative analysis of the fractional area of the intercellular junction as a percentage of the total capillary surface area, and that amounted to 0.2–0.4%. We believe that studies which assessed penetration of labeled protein or dextran into the glycocalyx over the cell body [26, 27] are not relevant to transendothelial permeability.

In support of our hypothesis, Curry and Adamson have published a review of the endothelial glycocalyx as a permeability barrier and mechano-sensor [41]. In their analysis of the glycocalyx thickness *vs*. barrier function with respect to both measured and modeled values for hydraulic conductivity, the authors state:

“*Thus, as argued by Weinbaum and colleagues *[40]*, measured L*_*p*_* values constrain estimates of surface layer thickness to a relatively narrow band, close to 100-150 nm”* and conclude that *“…at least part of the structure of the surface layer in the vicinity of the junctions which are the main water pathways differ from that across of most of the endothelial surface”*.

This means that the glycocalyx overlying the junctional cleft is either absent or structurally and functionally very different from the glycocalyx residing over the majority of the cell surface (Fig. 1). This conclusion also challenges the Revised Starling Principle, which describes a molecular filter that produces a protein-free filtrate in the sub-glycocalyx region that strongly restricts fluid movements from tissue to plasma [42]. Physiological findings questioning the Revised Starling Principle include the well-documented process of transcapillary refill [43] and that the oncotic properties of 20% albumin do recruit extravascular fluid [44–46].

The small area of the junction also has implications for studies examining glycocalyx shedding. The non-junctional area of the cell accounts for as much as 99.6–99.8% of the total surface area. Thus, in studies measuring shed glycocalyx biomarkers into the plasma [47] the vast majority of the shed biomarkers must originate from non-junctional locations and, therefore, are unlikely to contribute to changes in permeability. In direct support of this idea, Ince et al*.* demonstrated that significant increases in plasma glycocalyx biomarkers were not associated with increased vascular permeability. In a model of non-traumatic hemorrhagic shock, increases in heparan sulfate, syndecan-1 and hyaluronic acid were observed [48]. However, despite their use of four independent vascular permeability assays (plasma decay fluorescent dextran markers; Evans blue albumin loss; tissue wet/dry weight ratio; and intravital microscopy) they found no increase in endothelial permeability.

In a subsequent study, acute hemodilution with hydroxyethyl starch, balanced crystalloid or normal saline was associated with a graded increase in plasma syndecan-1 concentrations [49]. In these studies, there was no measurable increase in the vascular leakage of albumin or dextran (500 kDa) compared to controls. Collectively, these two investigations, using two very different conditions, provide compelling evidence that glycocalyx degradation is not associated with changes in vascular permeability. We identified only one study, in rats, where the sequences of events (shock, elevated glycocalyx shedding products, reduced glycocalyx thickness, increased capillary leakage) were correlated in a coherent way [50]. However, the effect of hemorrhagic shock on junctional permeability was not addressed in this study, so the pathway associated with measured increased albumin leakage could not be determined.

Lastly, recent evidence demonstrates that breakdown products of the glycocalyx have biological activity during inflammatory states. Yuan and colleagues demonstrated that thrombin-generated cleavage products of syndecan-3 and syndecan-4 increased lung microvascular albumin extravasation in the mouse lung [51]. It has been well established that heparan sulfate fragments bind to danger-associated molecular patterns (DAMPs) to amplify inflammation [52]. Thus, any study that induces glycocalyx degradation could be generating biologically active compounds that increase junctional permeability.

## Glycocalyx and capillary permeability in humans

There is sparse evidence that injury to the glycocalyx increases the capillary permeability for macromolecules in humans. Several approaches to the issue have been used, but each of them has limitation toward demonstrating causality.

1. Hundreds of studies have been published where *fragments of glycocalyx constituents*, such as syndecan-1 and heparan sulfate, have been measured in peripheral blood and increased plasma concentrations have been regarded as evidence of endothelial damage [47]. These proteins may arise from the vascular endothelium, but they are expressed on the many other tissues as well [53]. No study of their origin when appearing in high plasma concentrations after trauma and surgery has yet been performed. The plasma concentrations of these fragments reflect overall injury and outcomes, but the relationship between shedding and permeability seems to be weak.

2. *Structural measurements* of the glycocalyx can be performed by imaging the microcirculation, typically in the sublingual area [54]. Handheld imaging systems are commercially available for this purpose. They measure displacement of red blood cells to the center of the capillaries and define an exclusion zone called “perfused boundary region” that represents the glycocalyx thickness. We have reported that most studies of diabetes and all studies of major surgery show thinning of the glycocalyx layer as evidence of breakup of the glycocalyx. Fifteen studies measured both glycocalyx thickness by microimaging and circulating glycocalyx fragments in the plasma, but only 9 of them (60%) showed a correlation between the two variables [47]. This is marginally better than chance.

3. *Functional studies* quantified capillary leakage of macromolecules and intravascular fluid in settings where the glycocalyx is assumed or known to be damaged. If the glycocalyx functions as a permeability barrier, the intravascular half-life of exogenous albumin contained in an infusion of 20% albumin would be expected to be shortened by surgery and inflammation. However, postoperative patients and post-burn patients with moderately severe inflammation (plasma C-reactive protein of 60–80 mg/L) and patients undergoing surgery with minor hemorrhage had the same intravascular half-life of exogenous albumin when compared with healthy volunteers [55]. The post-burn patients given 20% albumin even showed a normal capillary leakage of albumin and fluid despite major elevations, even up to 100-fold, of plasma syndecan-1 [56].

Studies that provide a surplus of albumin are hampered by the possibility that a rise in plasma albumin blocks capillary leakage, which has been proposed in animals [57]. Margarson and Soni used hemoglobin changes to show that 79% of an infused albumin mass remained in the blood after 4 h in volunteers, while the corresponding amount in septic patients was 69% [44]. However, doubling the plasma albumin concentration in hypoalbuminemic patients with *septic shock* did not change the capillary leakage rate of radio-labeled albumin, which remained normal at 6.5% of the plasma pool per hour [58].

The use albumin as biomarker of capillary leakage is prone to errors in complex clinical situations [55], but a large body of evidence supports that both glycocalyx degradation products [59] and capillary leakage of albumin and fluid [60, 61] are increased in septic patients. Fleck et al*.* even reported quadrupled capillary leakage rates in patients with septic shock [62]. However, it is still difficult to determine the role of the glycocalyx in this process, as < 1% of the glycocalyx covers the endothelial junctions where albumin likely passes. Cleavage of other proteins specifically located in these junctions is an alternative possibility [63].

Capillary leakage of albumin during major surgery has both been reported as accelerated [64] and normal [65], but measurements performed *after* surgery are mostly normal [66–68]. Albuminuria occurs within 2–3 h of surgery [69], which is evidence of increased glomerular permeability; however, whether this is due to glycocalyx shedding is unclear. By contrast, chronic albuminuria in rats coexists with loss of glycocalyx [70].

**Fig. 2:**
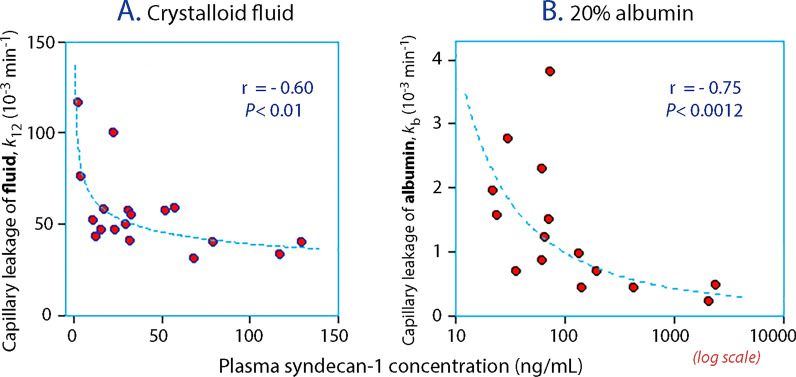
Inverse relationship between the plasma syndecan-1 concentration and the rate constant for capillary leakage of **A** infused fluid when 1.5 L of crystalloid solution was administered to males with a mean age of 72 years, and **B** albumin when 3 mL/kg of 20% albumin was infused in post-burn patients. Each point represents one infusion experiment. The kinetic constants (*k*_12_ and *k*_b_) were generated by volume kinetic analysis. Subplot **A** is derived from Reference [71], and subplot **B** was created based on data published in Reference [56]. Regressions were based on square-root-transformed plasma syndecan-1 concentrations measured (by the same laboratory) just before the infusions were initiated. Note the logarithmic scale in subplot **B**

Three studies using volume kinetic analysis have quantified capillary leakage of albumin and/or intravascular fluid in relation to plasma syndecan-1 concentrations and/or heparan sulfate concentrations in humans. All three trials refute that capillary leakage increases when shedding products in plasma are elevated. They suggest that the correlation between capillary leakage and glycocalyx degradation products is inverse, while a positive correlation would be expected if shedding increased permeability. The first two studies involved infusion of Ringer’s solution in elderly men [71] (Fig. 2A) and in patients with inflammation due to cholecystitis and appendicitis [72]. The third study administered 20% albumin to post-burn patients who had dramatically increased plasma concentrations of both syndecan-1 and heparan sulfate [56] (Fig. 2B). The reason for the inverse correlation is unclear. Possibilities include that higher concentrations of glycocalyx fragments are coupled with greater release inflammatory biomarkers, which raises the oncotic pressure [73]. A pharmacological effect of the shedding substances cannot be excluded, although other studies suggest that these fragments rather increase the capillary permeability [51, 52].

In conclusion, we have summarized preclinical studies that give the framework for the current view of the glycocalyx layer as a permeability barrier. We find difficulty to distinguish between the role of the glycocalyx and the endothelial junctions in this respect. Release of glycocalyx fragments to the plasma reflects injury and outcome, but the evidence for their role in capillary permeability in humans is weak.

## Data Availability

Not applicable.

## Abbreviations

AJ: Adherens junction
*L*_*p*_: Hydraulic conductivity
*P*: Transendothelial permeability coefficient
PV1: Plasmalemmal vesicle-associated protein-1 (a caveolae diaphragm protein)
